# Evaporation controls contact-dependent bacterial killing during surface-associated growth

**DOI:** 10.1093/ismeco/ycaf034

**Published:** 2025-02-21

**Authors:** Miao Han, Chujin Ruan, Gang Wang, David R Johnson

**Affiliations:** College of Land Science and Technology, China Agricultural University, Beijing 100193, China; Department of Environmental Microbiology, Swiss Federal Institute of Aquatic Science and Technology (Eawag), 8600 Dübendorf, Switzerland; Department of Environmental Microbiology, Swiss Federal Institute of Aquatic Science and Technology (Eawag), 8600 Dübendorf, Switzerland; College of Land Science and Technology, China Agricultural University, Beijing 100193, China; Department of Environmental Microbiology, Swiss Federal Institute of Aquatic Science and Technology (Eawag), 8600 Dübendorf, Switzerland; Institute of Ecology and Evolution, University of Bern, 3012 Bern, Switzerland

**Keywords:** bacterial interactions, contact-dependent killing, antagonism, T6SS, evaporation, *Vibrio cholerae*

## Abstract

Many bacteria employ contact-dependent killing mechanisms, which require direct physical contact with a target cell, to gain an advantage over competitors. Here, we hypothesize that evaporation-induced fluid flows determine the number of contacts between attacking and target cells, thus controlling killing efficacy. To test this, we experimentally manipulated the strength of the coffee ring effect (CRE) and measured the consequences on killing mediated by the type VI secretion system (T6SS). The CRE is caused by evaporation-induced fluid flows that move water and cells from the center to the periphery of a liquid droplet, consequently concentrating cells at the periphery. We found that the CRE significantly increases the number of contacts between attacking (*Vibrio cholerae*) and target (*Escherichia coli*) cells and enhances the ability of *V. cholerae* to kill and out-compete *E. coli*. We corroborated our findings with individual-based computational simulations and demonstrated that increased cell densities at the droplet periphery caused by the CRE increase killing. We further found that the T6SS firing rate, lethal hit threshold, and lysis delay significantly affect killing when the CRE is strong. Our results underscore the importance of evaporation-induced fluid flows in shaping bacterial interactions and controlling competitive outcomes.

## Introduction

Bacteria often engage in intense competition for limited resources and space [1, 2]. Many Gram-negative bacteria, including pathogens such as *Vibrio cholerae*, employ contact-dependent killing mechanisms to gain an advantage over their competitors [3, 4]. For example, upon contact with a target cell, attacking cells can use the type VI secretion system (T6SS) to inject toxic effector proteins into the target cell [5]. These toxins can disrupt critical cellular processes, including membrane integrity and metabolic functions, and cause cell death [1, 6], thus providing the attacking cell with a competitive advantage over the target cell.

Because direct contact between an attacking and a target cell is essential for toxin delivery [7, 8], environmental processes that modulate the spatial distributions and proximities of attacking and target cells are likely important determinants of killing efficacy. Here, we hypothesize that evaporation-induced fluid flows, which are important in systems where moisture levels fluctuate (e.g. unsaturated soils, plant leaves, human skin) [9], can modulate the spatial distributions of attacking and target cells and, in turn, control killing efficacy.

Our hypothesis is based on basic hydrodynamic principles. As a liquid droplet evaporates, two major fluid flows can form, which we refer to as the coffee ring effect (CRE) and Marangoni convection (MC) [10–12]. The CRE occurs when liquid evaporates more rapidly at the droplet’s periphery than at its center, which generates capillary-driven water flows that transport suspended particles (e.g. cells) toward the droplet periphery [9]. This can result in a concentrated ring-like cell deposition pattern. In contrast, MC arises from surface tension gradients within the liquid droplet that generate inward flows toward the droplet center [13, 14], which can counteract the CRE and result in a more uniform cell deposition pattern. The relative strengths of these two fluid flows will determine the extent to which cells are concentrated at the droplet periphery.

**Figure 1:**
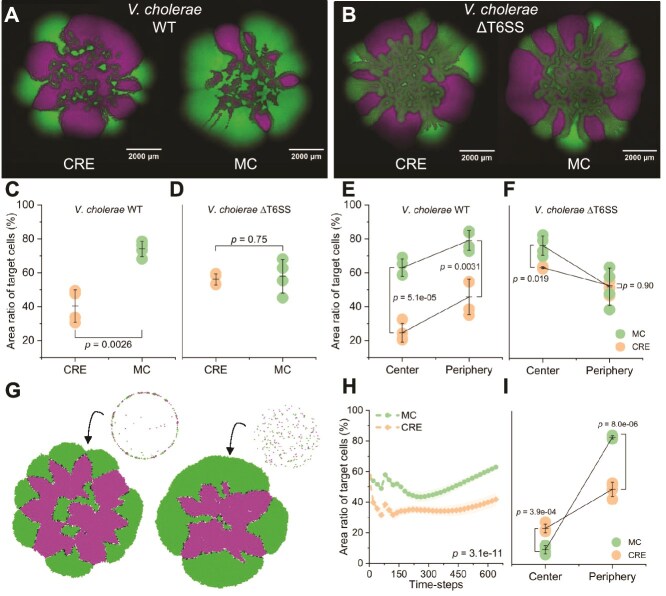
Effect of the CRE and MC on target strain distribution and survival. (A and B) Representative CLSM images of *Escherichia coli* TB204 target cells (green; initial concentration of 2 × 10^5^ cells ml^−1^) and *Vibrio cholerae* WT attacking cells (A) or *V. cholerae* ΔT6SS nonattacking cells (B) (magenta; initial concentration of 2 × 10^5^ initial cells ml^−1^) during surface-associated growth for CRE and MC conditions. (C and D) The ratio of the total area occupied by *E. coli* TB204 for CRE and MC conditions when cultured with (C) *V. cholerae* WT or (D) *V. cholerae* ΔT6SS at the end of the experiment. (E and F) The ratio of the area occupied by *E. coli* TB204 for CRE and MC conditions at the biomass center (4100 μm in diameter) or at the biomass periphery when cultured with (E) *V. cholerae* WT or (F) *V. cholerae* ΔT6SS. (G) Representative simulations of target cells (green) and attacking cells (magenta) during surface-associated growth for CRE and MC conditions. The cell positionings in the upper right are the initial positionings. The patterns on the lower left are the final spatial organizations after 640 time-steps. (H) The ratio of the total area occupied by target cells for CRE and MC conditions in the simulations as a function of simulation time-steps. The shaded regions are one standard deviation. (I) The ratio of the area occupied by target cells for CRE and MC conditions at the biomass center or at the biomass periphery in the simulations after 640 time-steps. The *P*-value in (H) is for a Mann–Whitney U test and all other *P*-values are for one-way ANOVA tests.

Because the relative strengths of the CRE and MC will determine the local densities of attacking and target cells across a surface, we expect that the relative strengths will also determine the efficacy of T6SS-mediated killing. As the CRE increases in relative strength and cells become more concentrated at the droplet periphery, the number of attacking cells in contact with target cells will increase, thus increasing killing. To test this, we conducted experiments with *V. cholerae* strain 2740-80 as the attacking strain (hereafter referred to as *V. cholerae* WT) [5] and *Escherichia coli* TB204 as the target strain [15]. To assess their spatial organization, we introduced the mCherry2 and green fluorescent protein-encoding genes into *V. cholerae* WT and *E. coli* TB204, respectively (see the Supplementary Information for details; note that we falsely colored mCherry2 to magenta in the images to improve visualization). We then performed surface-associated competition experiments by mixing *V. cholerae* WT (2 × 10^5^ cells ml^−1^) and *E. coli* TB204 (2 × 10^5^ cells ml^−1^) together and depositing 2-μl droplets of the mixture onto the centers of replicated lysogeny broth (LB) agar plates [15, 16] (note that the sizes of the cells are different, thus causing the initial area ratios to deviate from 50%). To impose CRE fluid flows, we directly inoculated the mixtures onto the LB agar plates (referred to as CRE conditions). To promote MC fluid flows, we added the nontoxic surfactant polyethylene glycol to the mixtures prior to inoculation onto the LB agar plates as described elsewhere (referred to as MC conditions) [10, 11]. We note that our application of the MC does not completely abolish the CRE; rather, it significantly reduces the accumulation of cells at the periphery when compared to CRE conditions (Supplementary Fig. S1).

**Figure 2:**
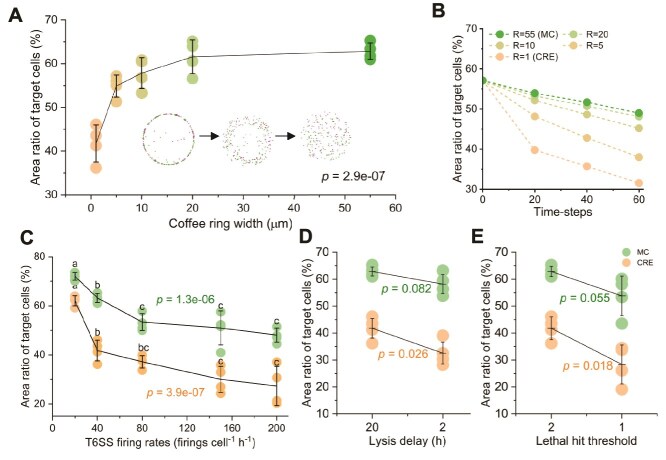
Effect of the strength of the CRE and T6SS parameters on the target strain. All data are from individual-based computational simulations with an initial cell ratio of 1:1 of target and attacking cells. (A) The ratio of the total area occupied by target cells after 640 time-steps as a function of the coffee ring width (CRW). The initial cell positions depicted at the bottom are for CRWs of 1, 20, and 55 μm. (B) The mean ratio of the total area occupied by target cells (*n* = 4) from 0–60 time-steps for CRWs between 1 and 55. (C) The ratio of the total area occupied by target cells for T6SS firing rates between 20 and 200 firings cell^−1^ h^−1^ for CRE and MC conditions after 640 simulation time-steps. (D) The ratio of the total area occupied by target cells for lysis delays of 20 or 2 h for CRE and MC conditions after 640 simulation time-steps. (E) The ratio of the total area occupied by target cells for lethal hit thresholds of 2 or 1 for CRE and MC conditions after 640 simulation time-steps. All *P*-values are for one-way ANOVA tests.

Using this approach, we found that the CRE significantly enhances the killing efficacy of *V. cholerae* WT (Fig. 1A and C). At the end of the experiment, the ratio of the total area occupied by *E. coli* TB204 was significantly lower for CRE conditions (40% ± 9.6%) than for MC conditions (74% ± 4.5%) (one-way ANOVA test; *P* = .0026, *n* = 4) (Fig. 1c). Moreover, when we analyzed the biomass centers and peripheries separately, the ratios of these local areas occupied by *E. coli* TB204 were both significantly lower for CRE conditions than for MC conditions (two-sample two-sided *t*-tests; *P* < .005, *n* = 4) (Fig. 1e). One might expect that *E. coli* TB204 should perform worse in the biomass center for MC conditions because the concentration of cells should be higher in the center when compared to CRE conditions, which is counter to what we observed (Fig. 1e). However, this expectation neglects the fact that the MC causes vigorous mixing of cells throughout the biomass area during evaporation [10], which could disrupt the cell contacts necessary for killing. We therefore find that CRE conditions improve the ability of *V. cholerae* WT to kill *E. coli* TB204 across the entire biomass area (Fig. 1C and E). We further tested whether the differential effects of the CRE and MC disappear as cell densities increase and found that this is indeed the case (Supplementary Fig. S2).

To quantitatively evaluate our main conclusion across a wider parameter space that is not experimentally tractable, we performed individual-based computational simulations with the CellModeller framework (see the Supplementary Information and Supplementary Tables S1 and S2 for details) [11, 17–19]. We simulated the CRE and MC conditions by varying the initial local cell densities, where the CRE concentrates cells at the droplet periphery and MC distributes them more evenly across the surface (Fig. 1G). We initiated simulations with equal numbers of target and attacking cells to match our experiments and generated spatial patterns that are qualitatively (Fig. 1A and G) and quantitatively (Fig. 1E and H) consistent with our experimental data. More precisely, the ratio of the total area occupied by target cells was significantly lower for CRE conditions (42% ± 4.2%) than for MC conditions (63% ± 11%) (Mann–Whitney U test; *P* = 3.1 × 10^−11^, *n* = 4) at the end of the simulations (Fig. 1H). As opposed to our experiments, we found that the ratio of the area occupied by *E. coli* TB204 in the biomass center was significantly larger for CRE conditions (23% ± 2.9%) than for MC conditions (9.2% ± 2.7%) (one-way ANOVA test; *P* = 3.9 × 10^−4^, *n* = 4) (Fig. 1I). However, our simulations did not consider the vigorous mixing that occurs during evaporation but only the spatial positions of cells after the evaporation process had ended. Nevertheless, our simulations clearly indicate that the additional cell contacts created by the CRE is a critical determinant of killing efficacy.

To investigate this further, we quantitatively described how the local cell density at the droplet periphery, which reflects the strength of the CRE, influences competitive outcomes (Fig. 2). We found that, as the width of the CRE decreases and cells become more concentrated along the droplet periphery, the ratio of the area occupied by target cells significantly decreases (one-way ANOVA test; *P* = 2.9 × 10^−7^, *n* = 4) (Fig. 2A and B; Supplementary Fig. S3). This further supports our conclusion that CRE-mediated cell contacts drive differences in contact-dependent killing efficacy. Thus, the relative strength of the CRE and MC is an important determinant affecting competitive outcomes.

We finally verified that the T6SS is essential to explain our experimental observations. To achieve this, we repeated our experiment with a mutant containing deletions in the *hcp1* and *hcp2* genes (referred to as *V. cholerae* ΔT6SS), which are essential for T6SS killing [20]. We did not observe a significant difference in the ratios of the total areas occupied by *E. coli* TB204 between the CRE and MC conditions (one-way ANOVA test, *P* = .75, *n* = 4) (Fig. 1B and D), demonstrating that a functional T6SS is essential to explain our results. When we analyzed the biomass periphery separately, we also did not observe a significant difference (one-way ANOVA test; *P* = .90, *n* = 4) (Fig. 1F). Together, these results demonstrate that a functional T6SS is essential to explain our results.

To further investigate the effects of T6SS and its properties, including properties that could be under regulatory control, we incorporated key parameters of T6SS functioning (i.e. firing rates, lethal hit threshold, and lysis delay) into our individual-based computational model (see the Supplementary Information for details). We found that the ratio of the area occupied by target cells decreases as the T6SS firing rate increases for both CRE (one-way ANOVA test, *P* = 3.9 × 10^−7^, *n* = 4) and MC (one-way ANOVA test, *P* = 1.3 × 10^−6^, *n* = 4) conditions (Fig. 2C; Supplementary Fig. S4). We observed less consistent effects for the lysis delay (time for toxin-induced lysis) (Fig. 2D; Supplementary Fig. S5) and the lethal hit threshold (number of needle strikes required to kill a target cell) (Fig. 2E; Supplementary Fig. S6). The ratio of the area occupied by target cells significantly decreases for longer lysis delays and smaller lethal hit thresholds for CRE conditions but has no effect for MC conditions. This is expected because, as there are fewer cell contacts for MC conditions, T6SS parameters will have smaller effects. Together, our results provide evidence that T6SS is the main determinant of our experimental observations.

In conclusion, our study demonstrates that evaporation-induced fluid flows can regulate cell contact-dependent killing and interspecies competition. We show that strong CRE leads to higher local cell densities and more contacts between attacking and target cells, consequently increasing the efficacy of contact-dependent killing. By systematically varying the relative strengths of these fluid flows and T6SS parameters, including firing rates, lethal hit thresholds, and lysis delay, we demonstrate their significance for competitive outcomes, particularly for CRE conditions. These findings highlight the importance of hydrodynamic processes in determining competitive outcomes and reveal potential strategies to manage microbial communities by rational manipulation of fluid flows.

## Supplementary Material

20250127_Han_SI_R1_ycaf034

## Data Availability

All data and code generated in this study have been deposited in the Eawag Research Data Institutional Collection (https://opendata.eawag.ch/) and are freely available to the public at https://doi.org/10.25678/000DFR. All microbial strains are available from the corresponding author upon request.
