# Supplementary material for: Evaporation controls contact-dependent bacterial killing during surface-associated growth
Source: ISME Commun. 2025 Feb 21;5(1):ycaf034. doi: 10.1093/ismeco/ycaf034 (PMC11922317; doi:10.1093/ismeco/ycaf034)
Supplement: 20250127_Han_SI_R1_ycaf034 [file 20250127_han_si_r1_ycaf034.pdf]

Supplementary Information

TITLE: Evaporation controls contact-dependent bacterial killing during surface-associated growth

RUNNING TITLE: Evaporation controls contact-dependent killing

AUTHORS: Miao Han[1, 2#], Chujin Ruan[2#], Gang Wang[1*], David R. Johnson[2, 3*]

AUTHOR AFFILIATIONS: [1]College of Land Science and Technology, China Agricultural University, Beijing, China; [2]Department of Environmental Microbiology, Swiss Federal Institute of Aquatic Science and Technology (Eawag), Dübendorf, Switzerland; [3]Institute of Ecology and Evolution, University of Bern, Bern, Switzerland. [#]These authors contributed equally.

[*]**CORRESPONDENCE:**

David R. Johnson: david.johnson@eawag.ch

Gang Wang: gangwang@cau.edu.cn

This file includes:

- Supplementary Materials and Methods
- Supplementary Tables S1 and S2
- Supplementary Figures S1-S6

Supplementary Materials and Methods

Bacterial strains and cultivation. We used the *lacZ*⁻ derivative of *Vibrio cholerae* 2740-80 as the parental strain, which is a non-toxicogenic El Tor strain isolated in 1980 from a patient in Florida, USA [1]. *V. cholerae* 2740-80 was initially constructed by deleting the *vipA* gene, followed by the construction of a carboxy (C)-terminal fusion between the *vipA* and *mCherry2* genes, with a DNA linker encoding 33Ala 33Gly serving as a separator [1]. This *VipA–mCherry2* fusion was used to complement the chromosomal in-frame deletion of *vipA*. We refer to this strain as *V. cholerae* wide-type (hereafter referred to as *V. cholerae* WT). The *V. cholerae* ΔT6SS strain was generated by introducing deletions in the *hcp1* and *hcp2* genes, which encode for the hemolysin-coregulated protein (Hcp) that is a key structural and functional component of the type VI secretion system (T6SS) [2]. We used *Escherichia coli* TB204 as the target strain, which is a derivative of strain MG1655 that constitutively expresses a green fluorescent protein-encoding gene from the lambda promoter located on the chromosome [3]. We grew all strains at 37 °C on lysogeny broth (LB) agar plates (10 g L^{-1} tryptone, 5 g L^{-1} yeast extract, 10 g L^{-1} NaCl, 1.5% agar, pH 7.4) or in liquid LB medium with shaking at 180 rpm.

Competition assays. We first incubated each strain individually overnight at 37 °C in liquid LB medium, washed the cells three times by centrifugation at 1000 × g for 5 min with fresh LB medium, and adjusted the cell concentration to 2×10^5 cells ml^{-1} of each strain or to 10^7 cells ml^{-1} of each strain as indicated in the main text. We then mixed *V. cholerae* with *E. coli* in a 1:1 volume ratio and deposited 2-μl aliquots of the mixture onto the centers of separate replicated LB plates. We imposed the coffee ring effect (CRE) by using the LB plates as described above and Marangoni convection (MC) by adding polyethylene glycol to the

bacterial mixture as we described previously [4, 5]. We previously demonstrated that polyethylene glycol has no effect on growth under our experimental conditions [4, 5]. We finally transferred the LB agar plates into a humidity-controlled environment (30% relative humidity) for 10 min at room temperature for droplet evaporation and then incubated the LB agar plates at 30 °C for 2 days.

Microscopy and image analysis. We acquired images of the resulting microbial biomass using a Leica TCS SP5 II CLSM (Leica Microsystems, Wetzlar, Germany) equipped with a 5x HCX FL objective, a numerical aperture of 0.12, and a frame size of 512 × 512 (resulting in a pixel size of 12.108 um). We used a laser emission of 488 nm for the excitation of green fluorescent protein and 514 nm for the excitation of red fluorescent protein. We set the emission filter to 519–551 nm for green fluorescent protein and 601–650 nm for red fluorescent protein. We performed quantitative analysis of fluorescent areas using ImageJ 1.52i (https://imagej.net) and calculated the ratio of areas occupied by *E. coli* by dividing the green fluorescent area by the total fluorescent area.

Initial spatial distributions of cells for CRE or MC conditions. We mixed *V. cholerae* (2×10^7 cells ml^{-1}) with *E. coli* (2×10^7 cells ml^{-1}) in a 1:1 ratio and deposited 2-μl aliquots of the mixtures onto the centers of separate replicated LB plates. We imposed CRE or MC conditions as described above. After evaporation, we imaged the initial cell distributions using the same CLSM as described above except we used a 20x HCX FL objective, a numerical aperture of 0.40, and a frame size of 1024 × 1024 (resulting in a pixel size of 0.758 um). We calculated the density of cells as the percentage of the total area occupied by cells within a rectangular region with dimensions of 250 μm × 40 μm.

Individual-based computational model. We used CellModeller as an agent-based computational modeling platform to simulate the effects of evaporation-induced fluid flows (including the CRE and MC) on the surface-associated growth of a mixture of attacking and target cells [5-8].

***Cell growth and division*.** We represented cells as rod-shaped entities with each cell acting as an independent agent whose behavior is influenced by its phenotype and interactions with neighboring cells. The model tracks individual cell growth, movement, and death. We designated attacking cells as magenta and target cells as green. A cell's volume (Vi) increases exponentially through elongation according to the equation $dV_i/dt = k_{grow,i}V_i$ from birth volume V_0, where dt is the simulation time-step and $k_{grow,i}$ is the cell growth rate. Once a cell reaches a volume $2V_0+\eta_{\text{division}}$, it divides lengthwise into two identical daughter cells. The term "η_{division}" adds uniform random noise to the division volume. Using CellModeller's 'collision detection and collision response' module, we modeled the physical expansion of cells based on cell-cell contacts, simulating cell shoving through computational geometry methods to resolve overlapping cell surfaces. This approach effectively captures spatial effects resulting from cell interactions, such as the random firing of T6SS and the subsequent death of target cells.

***Initial cell deposition patterns*.** CellModeller allows us to model surface-associated cell growth across a two-dimensional plane, which provides insights into initial cell deposition patterns that are difficult to test experimentally. To simulate the cell deposition patterns caused by the CRE, we set a narrow circular ring with a specific width as the initial deposition

area where cells are randomly distributed and oriented. In contrast, to simulate the MC, we set the ring width to be the entire droplet radius, allowing cells to be evenly distributed across the entire droplet surface. By varying the ring width, our model can flexibly replicate the impact of these two distinct physical phenomena on initial cell distributions.

T6SS firing and hit detection. In the model, we represent T6SS firing as discrete, spatially explicit events where each attacking cell fires toxin-laden needles from random points on its surface. The number of firings per time-step for each cell is drawn from a Poisson distribution with a defined mean firing rate. For each firing event, a needle of a specific length is generated, directed outward based on the local surface normal at the firing point. The model assumes that the length of the T6SS needle is proportional to the cell's radius, ensuring realistic firing dynamics. The firing process is constitutive and rapid, meaning it occurs frequently relative to the time-scale of cell movement, and each firing reduces the cell's growth rate to account for the metabolic cost of T6SS activity (termed “WeaponCost”). Following each firing event, the model checks whether the needle strikes another cell using computational geometry methods. This involves calculating the shortest distance between the needle vector and neighboring cells. If the needle intersects a neighboring cell, the hit is recorded.

Response to T6SS hits. Cells respond to T6SS hits based on a lethal hit threshold, meaning a cell dies after receiving a specific number of toxin injections. Each hit accumulates as part of a tally for each cell, and once the lethal hit threshold is reached, the affected cell changes color to black, indicating cell death. After a brief lysis delay, the cell then disappears from the simulation. The model also includes immunity mechanisms, where cells are immune to hits from cells of the same cell-type but vulnerable to hits from different cell-types.

The parameters of our model are summarized in Supplementary Tables S1 and S2. We simulated surface-associated cell growth from an initial randomly distributed 1:1 inoculum mixture. We stored the status and spatial location of each cell every 20 time-steps and we performed simulations until completing 640 time-steps.

Supplementary Tables

Supplementary Table S1: Model Parameters.

Category	Parameter	Symbol	Value [unit]	Source
Cells	Cell radius	radius	0.5 [μm]	[1, 6]
	Cell volume at birth (*Vibrio cholerae*)	initialVola	0.785 [μm^3]	[6]
	Cell volume at birth (*Escherichia coli*)	initialVolc	1.047 [μm^3]	[9]
	Max growth rate	cell.growthRate	1.0 [h^{-1}]	[6, 7]
	Cost of T6SS	WeaponCost	0.05 [-]	[7]
T6SS parameters	Max needles per cell	max_needles_per_cell	10 [-]	[7]
	Lethal hit thresholds	HitThresh	1–2 [-]	[7]
	Lysis delay	LysisDelay	2–20 [h^{-1}]	[7]
	T6SS firing rate	kFiring0	20–200 [firings $cell^{-1}$ h^{-1}]	[7, 9]
	Extracellular needle length	L_needle	0.5 [μm]	[7]
	Min. needle penetration for hit	L_penetration	10 [nm]	[7]
	Simulation time-step	dt	0.025 [h]	[7]
	Cell / needle sorting grid element size	h	10 [μm]	[7]
	CG absolute tolerance	ϵ_{CG}	0.001 [-]	[7]
	Max. contact iterations	M_{Iter}, max	8 [-]	[7]
	Regularization weight	α	0.04 (0.1 for chambers) [-]	[7]
	Growth restriction factor	1 / ɣ	0.002 (0.1 for chambers) [-]	[7]

Supplementary Table S2: Initial conditions for individual-based computational simulations of surface-associated growth.

Parameter	Units	Value
**T6SS killing**		
Lysis delay	h^{-1}	20
T6SS firing rate	firings $cell^{-1}$ h^{-1}	40
Lethal hit thresholds	-	2
**Cell deposition dominated by MC**		
Ring width	µm	55
Initial droplet radius	µm	55
Initial cell number	# cells	220
**Cell deposition dominated by CRE**		
Ring width	µm	1
Initial droplet radius	µm	55
Initial cell number	# cells	220

Supplementary Figures

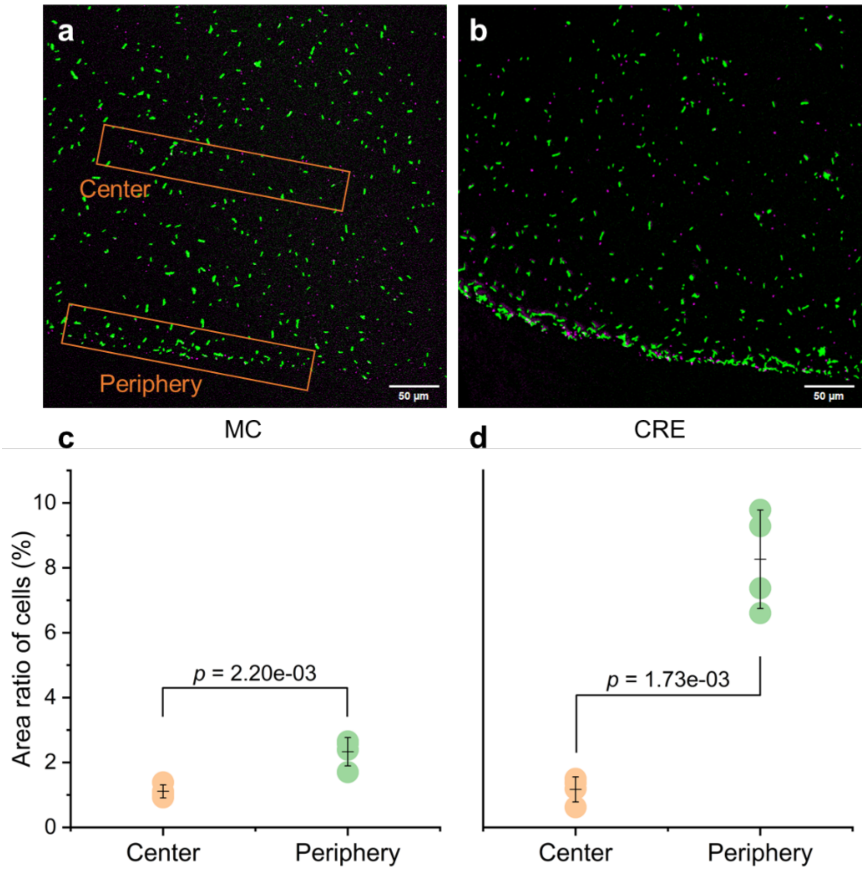

Supplementary Fig. S1: Initial spatial distributions of cells for CRE and MC conditions. a and **b** Representative CLSM images of the initial spatial distributions of *E. coli* TB204 target cells (green; initial concentration of 10^7 cells ml^{-1}) and *V. cholerae* WT attacking cells (magenta; initial concentration of 10^7 cells ml^{-1}) for (**a**) MC and (**b**) CRE conditions. **c** and **d** The ratio of the total area occupied by both *V. cholerae* WT and *E. coli* TB204 cells in a rectangular region with dimensions of 250 µm x 40 µm at the biomass center and periphery for (**c**) MC and (**d**) CRE conditions. The *p*-values are for one-way ANOVA tests.

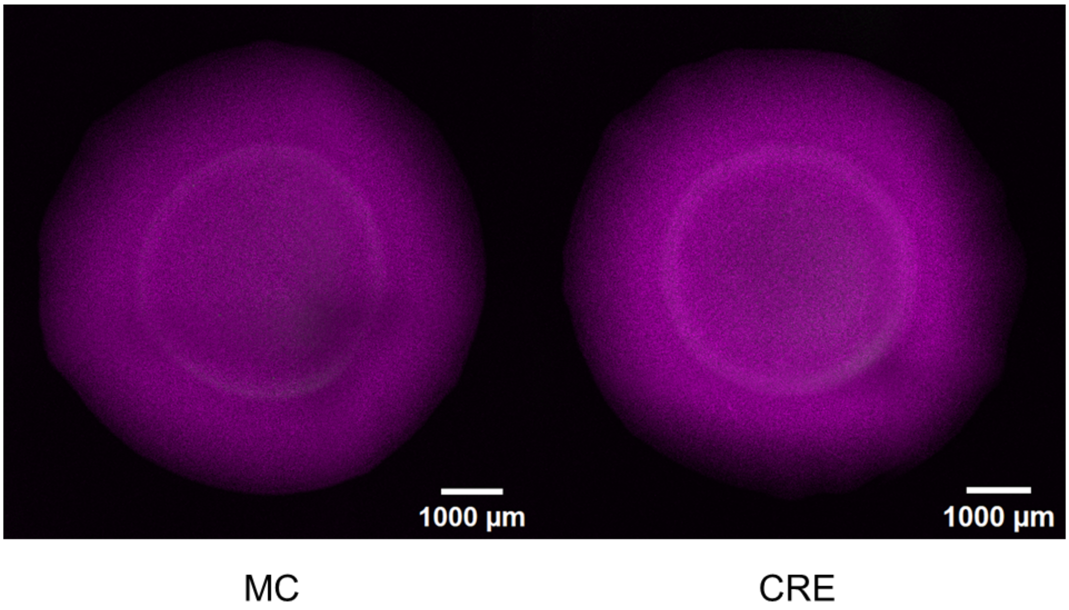

Supplementary Fig. S2: Effect of the CRE and MC at high initial cell densities. Representative CLSM images of *E. coli* TB204 target cells (green; initial concentration of 10^7 cells ml^{-1}) and *V. cholerae* WT attacking cells (magenta; initial concentration of 10^7 cells ml^{-1}) during surface-associated growth for CRE and MC conditions. Note that *E. coli* TB204 cells are no longer present for both CRE and MC conditions.

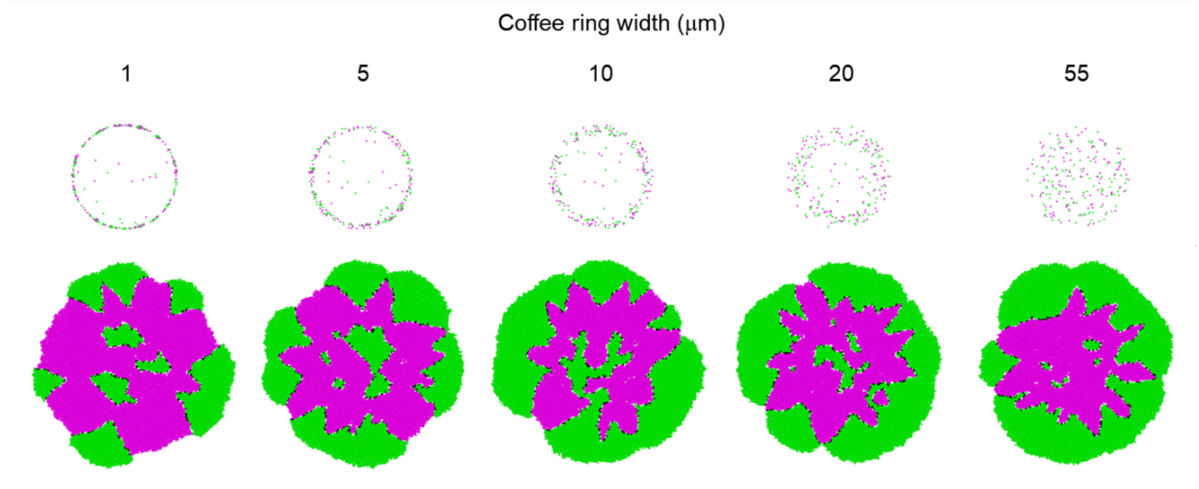

Supplementary Fig. S3: Simulations of the effect of the coffee ring width (CRW) on interspecific competition during surface-associated growth. The upper images are representative initial cell positionings while the lower images are representative simulations after 640 time-steps. The attacking cells are magenta and the target cells are green.

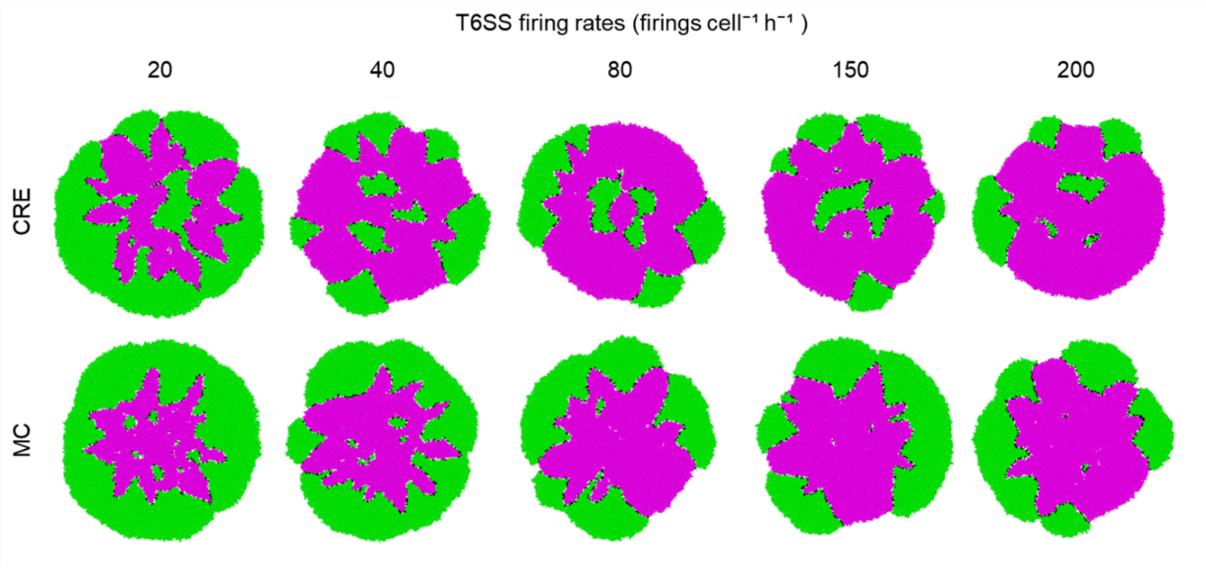

Supplementary Fig. S4: Simulations of the effect of the T6SS firing rate on interspecific competition during surface-associated growth. The upper images are representative simulations for CRE conditions and the lower images are representative simulations for MC conditions after 640 time-steps. The attacking cells are magenta and the target cells are green.

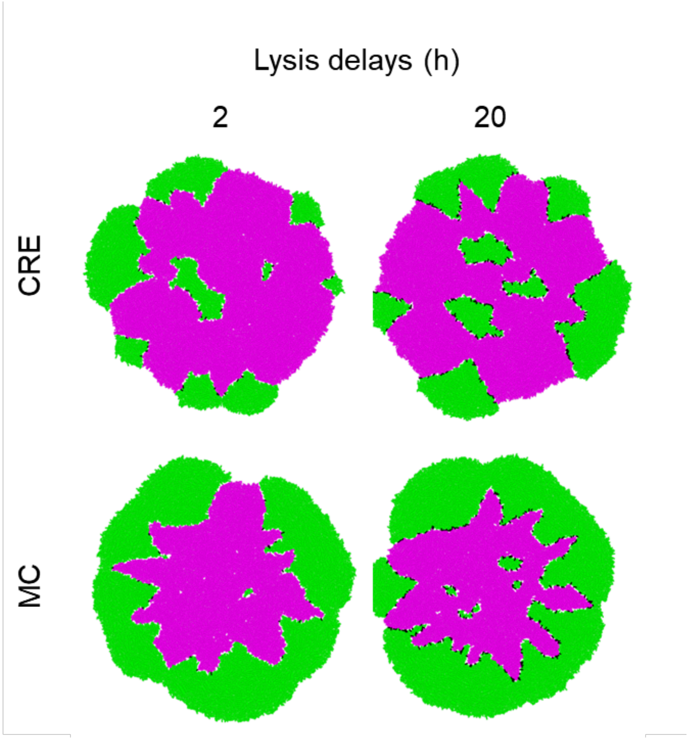

Supplementary Fig. S5: Simulations of the effect of the lysis delay on interspecific competition during surface-associated growth. The upper images are representative simulations for CRE conditions and the lower images are representative simulations for MC conditions after 640 time-steps. The attacking cells are magenta and the target cells are green.

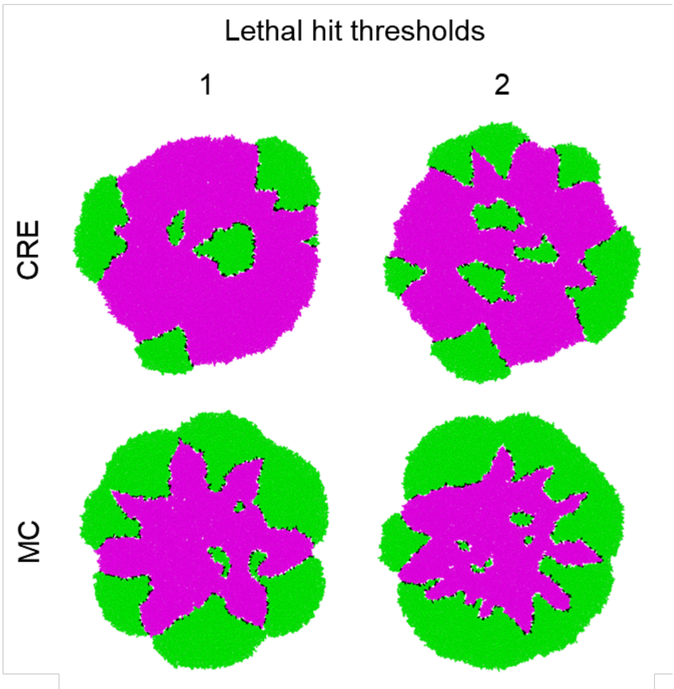

Supplementary Fig. S6: Effect of the lethal hit threshold on interspecific competition during surface-associated growth. The upper images are representative simulations for CRE conditions and the lower images are representative simulations for MC conditions after 640 time-steps. The attacking cells are magenta and the target cells are green.

Supplementary References

1. Basler M, Pilhofer M, Henderson GP, Jensen GJ, and Mekalanos JJ. Type VI secretion requires a dynamic contractile phage tail-like structure. Nature. 2012; 483:182-186.
2. Vettiger A, and Basler M. Type VI secretion system substrates are transferred and reused among sister cells. Cell. 2016; 167:99-110.
3. Ruan C, Ramoneda J, Kan A, Rudge TJ, Wang G, and Johnson DR. Phage predation accelerates the spread of plasmid-encoded antibiotic resistance. Nat Commun. 2024; 15:5397.
4. Ruan C, Ramoneda J, Chen G, Johnson DR, and Wang G. Evaporation-induced hydrodynamics promote conjugation-mediated plasmid transfer in microbial populations. ISME Commun. 2021; 1:54.
5. Ruan C, Borer B, Ramoneda J, Wang G, and Johnson DR. Evaporation-induced hydrodynamics control plasmid transfer during surface-associated microbial growth. npj Biofilms Microbi. 2023; 9:58.
6. Smith WPJ, Vettiger A, Winter J, Ryser T, Comstock LE, Basler M, et al. The evolution of the type VI secretion system as a disintegration weapon. PLoS Biol. 2020; 18:e3000720.
7. Smith WPJ, Brodmann M, Unterweger D, Davit Y, Comstock LE, Basler M, et al. The evolution of tit-for-tat in bacteria via the type VI secretion system. Nat Commun. 2020; 11:5395.
8. Booth SC, Smith WPJ, and Foster KR. The evolution of short- and long-range weapons for bacterial competition. Nat Ecol Evol. 2023; 7:2080-2091.
9. Otto SB, Servajean R, Lemopoulos A, Bitbol A-F, and Blokesch M. Interactions between pili affect the outcome of bacterial competition driven by the type VI secretion system. Curr Biol. 2024; 34:2403-2417.e2409.
